# Activin A regulates activation of mouse neutrophils by Smad3 signalling

**DOI:** 10.1098/rsob.160342

**Published:** 2017-05-17

**Authors:** Yan Qi, Jingyan Ge, Chunhui Ma, Na Wu, Xueling Cui, Zhonghui Liu

**Affiliations:** 1 Department of Immunology, College of Basic Medical Sciences, Jilin University, Changchun 130021, People's Republic of China; 2 Key Laboratory of Neuroimmunology, College of Basic Medical Sciences, Jilin University, Changchun 130021, People's Republic of China

**Keywords:** neutrophils, activin A, activin receptor, interleukin-6, Smad3

## Abstract

Activin A, a member of the transforming growth factor beta superfamily, acts as a pro-inflammatory factor in acute phase response, and influences the pathological progress of neutrophil-mediated disease. However, whether activin A can exert an effect on the activities of neutrophils remains unclear. In this study, we found that the release of activin A was enhanced from neutrophils of mouse when stimulated with lipopolysaccharide. Furthermore, neutrophils were not only the source of activin A but also the target cells in response to activin A, in which canonical activin signalling components existed, and levels of ACTRIIA, SMAD3 and p-SMAD3 proteins were elevated in activin A-treated neutrophils. Next, the role of activin A was determined in regulation of neutrophils activities. Our data revealed that activin A induced O_2_^−^ release and reactive oxygen species production, promoted IL-6 release, and enhanced phagocytosis, but failed to attract neutrophils migrating across the trans-well membrane. Moreover, we found that effect of activin A on IL-6 release from the peritoneal neutrophils of mouse was significantly attenuated by *in vivo Smad3* knockdown. In summary, these data demonstrate that activin A can exert an effect on neutrophils activation in an autocrine/paracrine manner through Smad3 signalling, suggesting that activin A is an important regulator of neutrophils.

## 1. Introduction

Activins are members of the transforming growth factor beta (TGF-β) superfamily, and have pleiotropic roles in physiological and pathological processes including regulation of embryogenesis, induction of mesoderm, protection of neurons, tumorigenesis and control of immune response [1–7]. So far, at least three different forms of activin have been identified, including activin A, activin B and activin AB [8,9]. Activin A has received the most attention until now, in part because it may play a crucial role in regulation of immune cells function [10].

Like most other TGF-β family members, activins share homology and canonical Smad-mediated signalling pathway with TGF-β. They conduce signalling by binding to type II activin receptor (ACTRII), forming a ligand/ACTRII complex, then the complex recruits type I activin receptor (ACTRI) to phosphorylates the recruited receptor-SMADs (SMAD2 and SMAD3) [11,12]. Once phosphorylated, SMAD2/3 dissociate from the receptor, bind to SMAD4, then the complex transmits the signal into the nucleus and promotes gene transcription [13–15].

Neutrophils are the most abundant white cell type in circulation that is recruited to the inflammatory sites rapidly in response to infection, injury and repair [16–18]. They have been established as the key mediator of multiple disease such as sepsis, acute respiratory distress syndrome (ARDS), rheumatoid arthritis, inflammatory bowel disease and different types of tissue injury [19,20]. Coincidentally, the strong expression of activin A has also been found in these diseases [6,21,22]. Several studies have demonstrated that activin A levels are elevated during sepsis, and serum concentrations of activin A in the patients who died from sepsis are higher than the survivors [23–25]. Additionally, the upregulated activin A is a pathogenic factor in the murine lung that causes a phenotype similar to ARDS [26]. Mounting evidence suggests that activin A may influence the pathological progress of neutrophil-mediated disease [22,27].

Thus, it is meaningful to perform a search to investigate the relationship between neutrophils and activin A. However, we know nothing about whether activin A can exert an effect on the activities of neutrophils until now. This study demonstrated for the first time that activin A could regulate neutrophil activities in an autocrine/paracrine manner.

## 2. Material and methods

### 2.1. Animals

Male C57BL/6 mice from 8 to 10 weeks were provided by the animal centre of Jilin University.

### 2.2. Reagents and antibodies

Activin A and APC-labelled mouse anti-ACTRIIA antibody were purchased from R&D Company. PE-labelled rat anti-Gr-1 antibody and FITC-conjugated mouse anti-CD11b antibody were provided by eBioscience Company. The fluoSpheres carboxylate-modified red fluorescent microspheres (1 µm diameter) were supplied by Invitrogen. Superoxide detection kits and reactive oxygen species (ROS) assay kits were obtained from Beyotime Company.

### 2.3. Isolation of the neutrophils

Peripheral blood neutrophils of mouse were isolated by fluorescence-activated cell sorting analysis (FACS). Briefly, fresh blood of mice was incubated with 6% dextran T-70 (1 : 4) at room temperature for 20 min, and then the leucocyte-rich upper fraction was collected. The cells were stained with PE-conjugated anti-Ly-6G and FITC-conjugated anti-CD11b antibodies or with appropriate fluorochrome-conjugated isotype IgG as control for 30 min. Neutrophils (CD11b^+^Ly-6G^+^) were sorted by FACS on BD FACSAria II.

The peritoneal neutrophils were prepared as described previously [23]. Briefly, 1 ml of 9% casein was injected into the peritoneal cavity per mouse, and after 24 h the same volume of casein was injected. Three hours later, peritoneal cells were collected and cultured in 10% fetal calf serum (FCS)-RPMI 1640 medium in 5% CO_2_ at 37°C to remove the peritoneal macrophages [24]. One hour later, suspending cells were collected, and neutrophils (CD11b^+^Ly-6G^+^) were sorted by FACS. Neutrophils were evaluated by cytology following diff-quick staining.

### 2.4. Immunofluorescent staining

The type IIA receptor of activin (ACTRIIA) and Ly-6G, a marker of mouse neutrophils, on neutrophils was examined by the dual imunofluorescent staining. In brief, the isolated neutrophils were incubated with rabbit anti-ACTRIIA antibody for 60 min. After being washed with phosphate-buffered saline (PBS) three times, the cells were also incubated with FITC-conjugated goat anti-rabbit IgG antibody for 30 min, and then incubated with PE-conjugated anti-Ly-6G antibody for 30 min. Finally, the cells were observed under a fluorescence microscope.

### 2.5. RT-PCR

Total RNA from neutrophils was extracted using the TRIzol reagent according to the manufacturer's protocol (Invitrogen). PCR was performed using the one-step RT-PCR kit according to the manufacturer's instructions (Takara Biotechnology Co). PCR products were subjected to 1.5% agarose gel electrophoresis, and the specific bands were analysed using ImageMaster VDS (Pharmacia Biotech Company). Primer sequences are available in table 1.

**Table 1.** Primer sequences.

target	primers	sequences	products size (bp)	GenBank no.
*Gapdh*	sense	5′-GACTTCAACAGCAACTCCCACTC-3′	107	BC083149
	antisense	3′-TAGCCGTATTCATTGTCATACCAG-5′		
*Activin βA*	sense	5′-GAGAGGAGTGAACTGTTGCT-3′	514	NM_008380
	antisense	3′-ATGACTGTTGAGTGGAAGGA-5′		
*ActRIIA*	sense	5′-ATTGGCCAGCATCCATCTCTTG-3′	296	XM_123799
	antisense	3′-GCCACCATCATAGACTAGATTC-5′		
*ActRIIB*	sense	5′-TGCTGAAGAGCGACCTCAC-3′	544	NM_007397
	antisense	3′-AGCAGGTCCACATTGGTGAC-5′		
*Smad3*	sense	5′-CCAGCACACAATAACTTGGA-3′	574	NM_016769
	antisense	3′-AGACACACTGGAACAGCGGA-5′		

### 2.6. Detection of activin A

The peritoneal neutrophils of 1 × 10^6^/well were incubated in the absence or the presence of 200 ng ml^−1^ lipopolysaccharide (LPS) for 2–12 h. The supernatants of the cultured neutrophils were collected, and activin A levels were detected using enzyme-linked immunosorbent assay (ELISA) kit according to the manufacturer's protocol (R&D).

### 2.7. Western blotting

The neutrophils of 2 × 10^6^/well were incubated in the absence or the presence of activin A for 2 h. The cells were lysed in protein lysis buffer, and the lysate was cleared by centrifugation at 10 000 r.p.m. min^−1^ for 20 min. The proteins in the supernatant were separated by SDS-PAGE and transferred onto a polyvinylidene difluoride membrane. The membrane was probed with anti-ACTRIIA, SMAD3, phosphorylated SMAD3 (p-SMAD3) and GAPDH antibodies, respectively. Finally, the labelled proteins were detected by chemiluminescence (ECL-Plus; Amersham Pharmacia Biotech).

### 2.8. Analysis of intracellular ROS

Intracellular ROS was detected by fluorescence probe DCFH-DA. DCFH-DA itself is without fluorescence, which can be hydrolysed by ester hydrolysis enzyme to DCFH inside the cells. ROS can oxidize DCFH without fluorescence into DCF with fluorescence. So the levels of intracellular ROS can be detected by measuring fluorescence intensity of DCF. The isolated neutrophils were incubating with DCFH-DA in a humidified incubator containing 5% CO_2_ at 37°C for 20 min. After incubation, 0–10 ng ml^−1^ activin A or 200 ng ml^−1^ LPS were added, and the increase of fluorescence was measured by flow cytometry.

### 2.9. Detection of superoxide

Superoxide (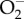) that can decompose water-soluble tetrazolium salt WST-1 was detected with superoxide detection kits (Beyotime, China). Briefly, the neutrophils were stimulated with activin A or LPS in 5% CO_2_ at 37°C for 12 h. After discarding the supernatant, the cells were incubated with WST-1 in dark at 37°C for 5 min. Absorbance was detected at 450 nm by an automated microtitre plate reader.

### 2.10. Detection of IL-6 and TNF-α

The supernatants of the cultured neutrophils in the presence or absence of activin A were collected, and levels of interleukin-6 (IL-6) and tumour necrosis factor-α (TNF-α) were detected by ELISA kit according to the manufacturer's protocol (eBioscience).

### 2.11. Analysis of phagocytosis of neutrophils

The neutrophils of 1 × 10^6^/well were treated with activin A or LPS in 5% FCS/RPMI 1640 medium in 5% CO_2_ at 37°C for 12 h, and then carboxylate-modified fluorescent microspheres with red fluorescence were added for 1 h. Neutrophils were rinsed with PBS, and then the ratio of phagocytosis was examined with flow cytometry.

### 2.12. Neutrophil chemotaxis assay

Neutrophil chemotaxis was determined using trans-well chambers (3 µm pore size, Corning). Briefly, the neutrophils were incubated with 1 µmol l^−1^ CFSE at 37°C for 10 min. The neutrophils (1 × 10^6^/well) labelled by CFSE with green fluorescence were loaded into the upper chamber and the lower chamber was full of 5% FCS–RPMI 1640 medium containing activin A or FMLP (Sigma) as positive control. The cells were cultured at 37°C in 5% CO_2_ for 45 min. The number of cells that migrated to the underside of the membrane was counted under inverted fluorescence microscope.

### 2.13. *Smad3* knockdown

*Smad3* was knocked down *in vivo* with pGCsi-U6/Neo-*Smad3* shRNA as described previously [28]. Briefly, 1 ml of 9% casein was injected into peritoneal cavity per mouse. After 24 h, the equivalent volume of casein was injected. 2 h later, each mouse was injected intraperitoneally with 3 µg pGCsi-U6/Neo-*Smad3* shRNA-lipofectamine 2000 reagent complex in accordance with the manufacturer's protocol (Invitrogen) or with 3 µg pGCsi-U6/Neo-lipofectamine 2000 reagent complex as empty plasmid control. After 12 h, neutrophils were isolated from peritoneal cells and incubated in the presence or absence of activin A for 12 h, and then the supernatants were collected and IL-6 levels were examined by ELISA.

### 2.14. Statistical analysis

All data are expressed as means ± s.d. The data were analysed using a Student's *t*-test, and values of *p* < 0.05 were considered statistically significant.

## 3. Results

### 3.1. LPS promotes the release of activin A from neutrophils

**Figure 1.**
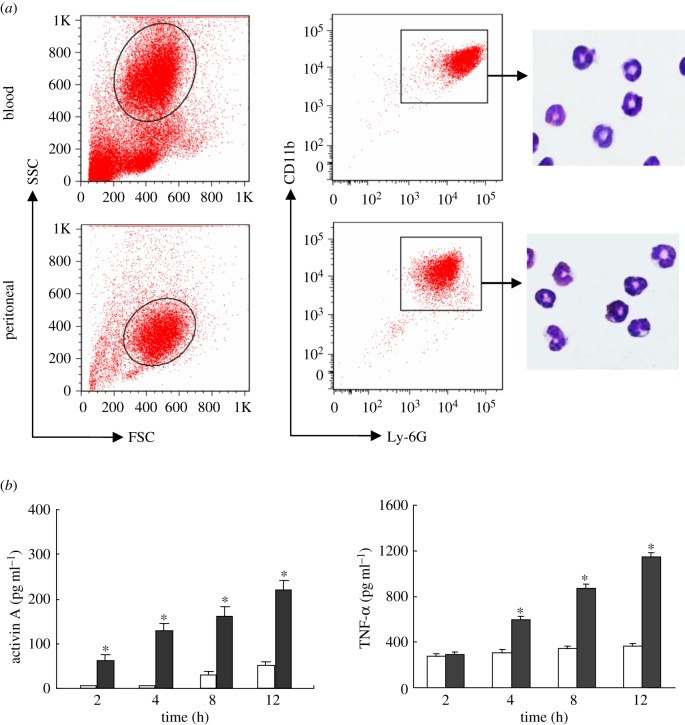
Release of activin A from mouse neutrophils treated with LPS. (*a*) CD11b^+^Ly-6G^+^ cells in peritoneal and peripheral blood cells of mouse were sorted by FACS, respectively, and assessed by cytology following diff-quick staining. (*b*) The levels of activin A and TNF-α in the supernatant of the cultured peritoneal neutrophils treated with 200 ng ml^−1^ LPS were examined by ELISA. Open bar, culture medium control; filled bar, LPS.

CD11b and Ly-6G are often used in combination to identify mouse neutrophils, thus we sorted CD11b^+^Ly-6G^+^ cells from peritoneal or peripheral blood cells with greater than 99.5% purity by FACS. The isolated cells which stained with diff-quick staining reagent had the typical neutrophil feature of the ring- and lobe-shaped nuclei (figure 1*a*). We know that LPS can induce neutrophil activation, and TNF-α can stimulate human neutrophils to release activin A [29,30]. In this study, neutrophils were stimulated with LPS as agonist, and TNF-α and actvin A production were measured by ELISA. The result showed that LPS not only promoted the release of TNF-α from neutrophils, but also enhanced the production of activin A (figure 1*b*).

### 3.2. Canonical activin signalling components exist in mouse neutrophils

**Figure 2.**
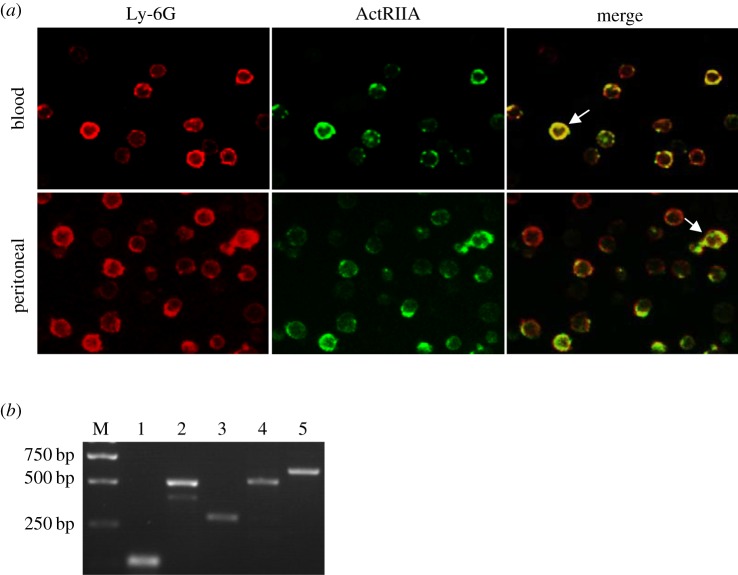
Expression of activin signalling components in mouse neutrophils. (*a*) Expression of ActRIIA on peritoneal or peripheral blood neutrophils was examined by dual immunofluorescent staining with anti-Ly-6G antibody (red) and anti-ActRIIA antibody (green). The yellow represented the superposition of ActRIIA and Ly-6G on neutrophils (merge). (*b*) The expression of *Activin βA*, *ActRIIA*, *ActRIIB* and *Smad3* mRNA in the peritoneal nertrophils of mouse was examined by RT-PCR. M, molecular weight marker (bp); lane 1, *Gapdh* (107 bp); lane 2, *Activin βA* (514 bp); lane 3, *ActRIIA* (296 bp); lane 4, *ActRIIB* (544 bp); lane 5, *Smad3* (574 bp). *p < 0.01, compared with control group.

To investigate whether neutrophils are able to sense activin A, we examined the expression of activin receptor by immunofluorescent staining. The results showed that Ly-6G and ACTRIIA were co-expressed on the isolated peritoneal and peripheral blood neutrophils (figure 2*a*). Next, the mRNA expressions of activin signalling components were measured in peritoneal neutrophils by RT-PCR. The results revealed that not only *Activin βA*, but also *ActRIIA*, *ActRIIB* and *Smad3* mRNA were expressed in peritoneal neutrophils of mouse (figure 2*b*). Thus, canonical activin signalling components exist in mouse neutrophils, indicating that activin A may act on neutrophils.

### 3.3. Activin A modulates activities of mouse neutrophils

**Figure 3.**
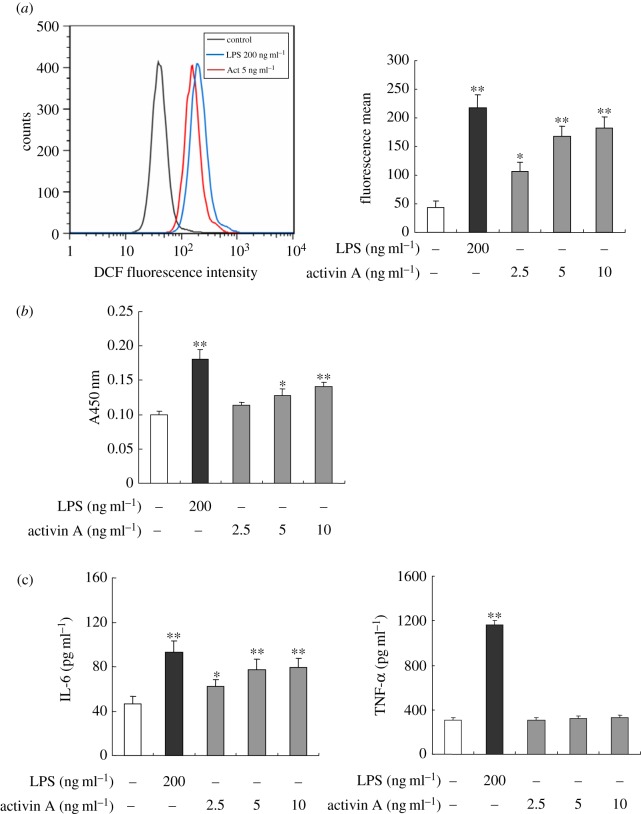
Effects of activin A on respiratory burst and IL-6 release of mouse neutrophils. (*a*) The peritoneal neutrophils of mouse were incubated for 30 min in the absence or presence of activin A and LPS, and then the intracellular ROS was detected using fluorescence probe DCFH-DA. Each bar represented the fluorescence mean from three independent experiments. *p < 0.05, **p < 0.01, compared with control group. (*b*) 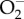 production was detected with superoxide detection kits. (*c*) The levels of IL-6 and TNF-α in the supernatant of the cultured neutrophils were assayed by ELISA. All values were presented as mean ± s.d. of three independent experiments (*n* = 6). *p < 0.05, **p < 0.01, compared with control group.

Neutrophils play a critical role in innate defence via their primary functions, such as respiratory burst and the release of the pro-inflammatory cytokines [31,32]. To investigate whether neutrophils can change their behaviour in response to activin A, we first assessed the effects of activin A on respiratory burst of neutrophils. The respiratory burst generates ROS from neutrophils, which is responsible for killing the invading microbes. In this study, we found that activin A induced 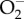 release and ROS production (figure 3*a*,*b*). Then, we evaluated the effects of activin A on IL-6 and TNF-α release from neutrophils. As shown in figure 3*c*, activin A promoted production of IL-6 in mouse neutrophils, but did not alter levels of TNF-α.

**Figure 4.**
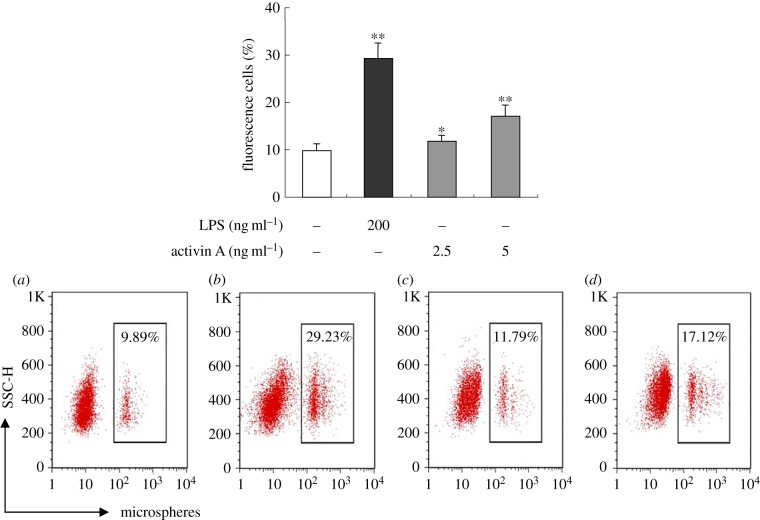
Effects of activin A on phagocytosis of mouse neutrophils. The peritoneal neutrophils of mouse were incubated in the absence or the presence of activin A and LPS for 12 h, and the phagocytic abilities of neutrophils to microspheres with red fluorescence were evaluated by flow cytometry. A representative experiment of the three performed is shown with (*a*) culture medium control, (*b*) 200 ng ml^−1^ LPS, (*c*) 2.5 ng ml^−1^ activin A and (*d*) 5 ng ml^−1^ activin A. The graph represents the phagocytic capabilities of neutrophils to microspheres with red fluorescence from three independent experiments. *p < 0.05, **p < 0.01, compared with control group.

Neutrophils also have phagocytic activities to kill the invading bacteria and other foreign matter. Thus, the ability of phagocytosis of neutrophils was examined by flow cytometry. Our data revealed that LPS as positive control could obviously promote phagocytosis of neutrophils to microspheres with red fluorescence, compared with a control group, and activin A also significantly enhanced phagocytic ability of neutrophils to microspheres with red fluorescence (figure 4).

**Figure 5.**
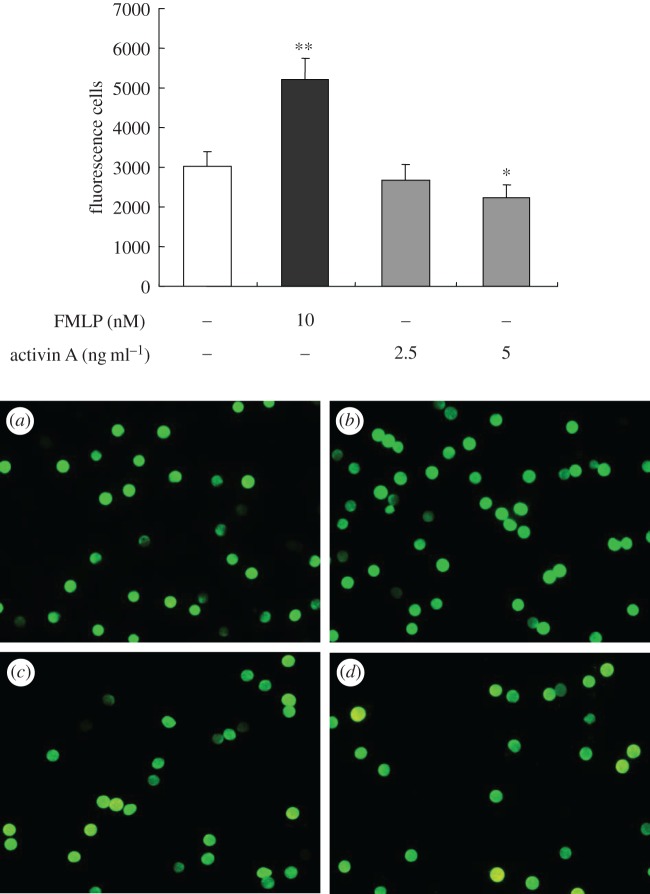
Assay of chemotaxis of mouse neutrophils treated with activin A. The migration of neutrophils labelled by CFSE with green fluorescence was determined using trans-well chambers and the number of cells that migrated to the underside of the membrane was counted under inverted fluorescence microscope. A representative experiment of the three performed is shown with (*a*) culture medium control, (*b*) 10 nmol l^−1^ FMLP, (*c*) 2.5 ng ml^−1^ activin A and (*d*) 5 ng ml^−1^ activin A. The graph represents the migrated neutrophils from three independent experiments. *p < 0.05, **p < 0.01, compared with control group.

Finally, chemokines are known to regulate neutrophil recruitment to sites of infection or injury [26,27]. Two-dimensional trans-well chambers were used to examine whether activin A could affect neutrophil chemotaxis *in vitro*. We found that the chemoattractant FMLP attract neutrophils migrating to the lower chamber, whereas addition of activin A to the lower chamber reduced the number of neutrophils that migrated through the membrane (figure 5). Collectively, these data suggest that activin A is an important mediator in the regulation of neutrophil activation, but is not a potential chemoattractant for neutrophils.

### 3.4. Activin A enhanced phosphorylation of SMAD3 in neutrophils

**Figure 6.**
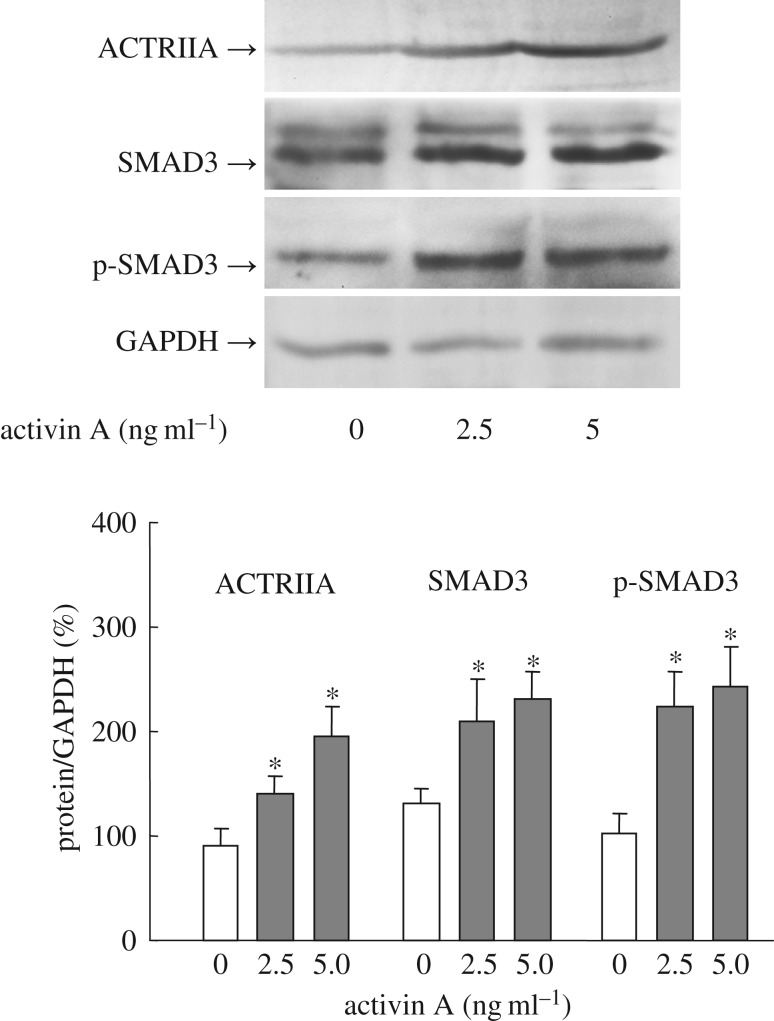
Expression of ACTRIIA and SMAD3 in mouse peritoneal neutrophils treated with activin A. The levels of ACTRIIA, SMAD3 and p-SMAD3 proteins in activin A-treated neutrophils were examined by western blotting. The graph represents relative protein levels from triplicate determinations. *p < 0.01, compared with control group.

Activin A combines activin receptors and activates the downstream signalling molecules SMAD3. In this study, the results revealed that ACTRIIA, SMAD3 and p-SMAD3 levels increased obviously in nertrophils stimulated by activin A (figure 6), suggesting that activin A may act as a regulator of neutrophils via ACTRIIA-SMAD3 signalling.

### 3.5. *Smad3* knockdown attenuated the effect of activin A on IL-6 release

**Figure 7.**
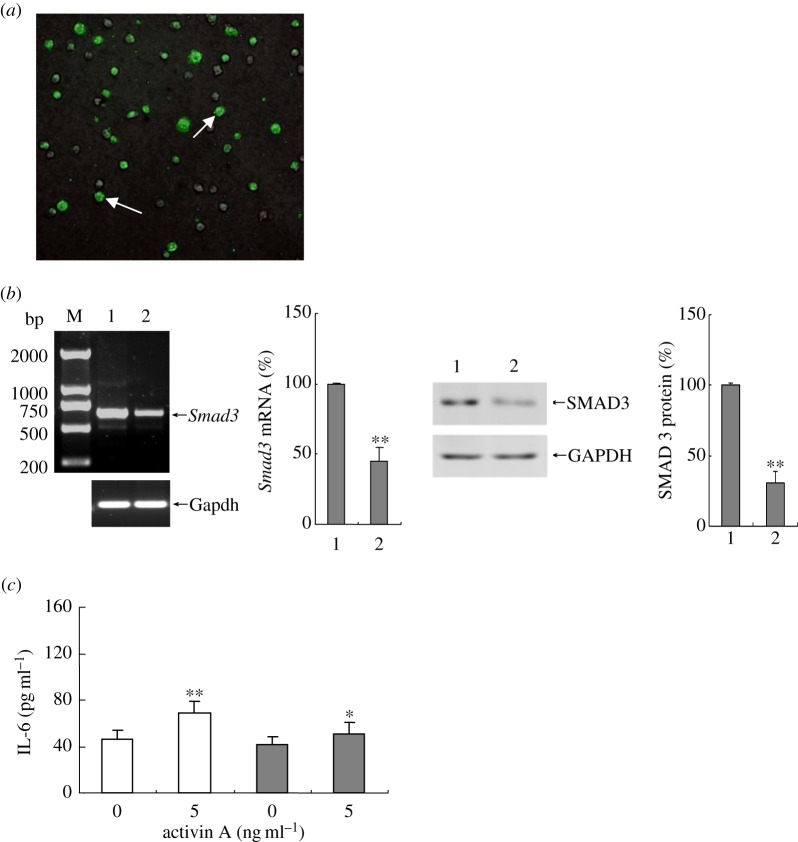
Assay of IL-6 production from *Smad3* knockdown neutrophils. (*a*) The isolated peritoneal neutrophils transfected *in vivo* with pGCsi-U6/Neo-GFP-*Smad3* shRNA (green) were observed under fluorescence microscope. (*b*) Expression of *Smad3* mRNA and SMAD3 protein in neutrophils transfected with pGCsi-U6/Neo (plasmid control) and pGCsi-U6/Neo-*Smad3* shRNA (*Smad3* shRNA) was examined by RT-PCR and western blotting. M, molecular weight marker (bp); lane 1, plasmid control; lane 2, *Smad3* shRNA. The graph represents the relative *Smad3* mRNA and SMDA3 protein levels from three independent experiments. **p < 0.01, compared with plasmid control. (*c*) IL-6 levels in the supernatant of the cultured plasmid control (open bar) and *Smad3* knock-down (filled bar) neutrophils were examined by ELISA. **p < 0.01, compared with 0 ng ml^−1^ activin A plasmid control group; *p < 0.05, compared with 5 ng ml^−1^ activin A plasmid control group.

To confirm Smad3 signalling mediated activin A action, the peritoneal neutrophils were transfected with pGCsi-U6/Neo-GFP-*Smad3* shRNA *in vivo* to knock down *Smad3* gene expression (figure 7*a*,*b*). Additionally, the results of this study revealed that the effect of activin A on IL-6 release was significantly weakened after knockdown of *Smad3* gene in neutrophils (figure 7*c*). These findings further demonstrated that activin A as regulator could activate neutrophils through Smad3 signalling.

## 4. Discussion

In this study, we found that activin A could be produced by LPS-stimulated neutrophils. More than that, activin A was able to regulate the functions of neutrophils, such as respiratory burst, IL-6 release, phagocytosis and migration. Furthermore, the effect of activin A on IL-6 release was significantly weakened after knockdown of *Smad3* in neutrophils. These data provide the evidence that activin A can regulate neutrophil activation in an autocrine/paracrine manner via Smad3 signalling.

Upregulated expression of activin A has been observed in various acute and chronic inflammations. The production of activin A can be stimulated by pro-inflammatory cytokines such as LPS, TNF-α and IL-1β [33–35]. The main source of activin A under inflammatory stimuli remains indeterminate, but various immune cells including monocytes, macrophages, mastocytes and neutrophils may be potential candidates [29,34,36–38]. These previous studies have reported that the cultured neutrophils secreted activin A *in vitro* when stimulated by TNF-α. Although it has also been claimed that LPS itself is insufficient to elicit activin A release from neutrophils *in vitro*, it significantly induced activin A mRNA expression [29,34]. In this study, we found that LPS were able to promote the release of activin A from neutrophils, further confirming that inflammatory stimuli, such as LPS and TNF-α can induce the release of activin A from neutrophils [29,34].

To investigate whether neutrophils are able to respond to activin A, we first examined the expression of activin signalling components. The results showed that not only activin receptor, but also other canonical activin signalling components, exist in mouse neutrophils. In addition, expressions of ACTRIIA, SMAD3 and p-SMAD3 proteins were elevated in activin A-treated neutrophils. Taken together, these data demonstrate that neutrophils not only produced activin A, but also were the target cells in response to activin A.

Next, to determine the role of activin A in regulation of neutrophil activities, respiratory burst, release of the pro-inflammatory cytokines and phagocytic ability of neutrophils were further analysed. We found that activin A induced O_2_^−^ release and ROS production, enhanced phagocytosis, and promoted IL-6 release in mouse neutrophils. Moreover, *Smad3* knockdown significantly attenuated effect of activin A on IL-6 release in neutrophil. These data indicate that activin A may induce neutrophil priming and play an important role in neutrophil activation via Smad3 signalling.

Interestingly, activin A is not a potential chemoattractant for neutrophils and addition of activin A to the lower chamber reduced the number of neutrophils that migrated through the membrane. Neutrophils migrate from the bloodstream to sites of infection or injury to kill the invading bacteria and other foreign matter, but the highly destructive capacity of neutrophils can also raise the potential to damage the host is healthy tissues [39,40]. Activin A has been generally accepted as a pro- and anti-inflammatory mediator depending on both cellular context and stage of disease [36,41–44]. It is possible that activin A plays a dual role in regulating neutrophil functions. We suppose that, on the one hand, activin A may induce the activation of neutrophils in the early phase of inflammation; on the other hand, the high level of activin A enriched at inflammatory sites might stop the development of inflammation by restricting the migration of neutrophils in the late phase of inflammation. But our study was limited to knowing the effects of activin A *in vivo*, so more comprehensive scrutiny of the effect of activin A in neutrophil-mediated disease, such as sepsis, ARDS, rheumatoid arthritis and different types of tissue injury, is highly warranted.

In summary, these data demonstrate that activin A can exert an effect on neutrophil activation in an autocrine/paracrine manner through Smad3 signalling, suggesting that activin A is an important regulator of neutrophils.

## References

[1] Namwanje M, Brown CW 2016 Activins and inhibins: roles in development, physiology, and disease. Cold Spring Harb. Perspect. Biol. 8, a021881 (doi:10.1101/cshperspect.a021881) 27328872 10.1101/cshperspect.a021881 PMC4930927

[2] van den Ameele J, Tiberi L, Bondue A, Paulissen C, Herpoel A, Iacovino M, Kyba M, Blanpain C, Vanderhaeghen P 2012 Eomesodermin induces Mesp1 expression and cardiac differentiation from embryonic stem cells in the absence of Activin. EMBO Rep. 13, 355–362. (doi:10.1038/embor.2012.23) 22402664 10.1038/embor.2012.23 PMC3321156

[3] Kaiser O, Paasche G, Stover T, Ernst S, Lenarz T, Kral A, Warnecke A 2013 TGF-beta superfamily member activin A acts with BDNF and erythropoietin to improve survival of spiral ganglion neurons *in vitro*. Neuropharmacology 75, 416–425. (doi:10.1016/j.neuropharm.2013.08.008) 23973291 10.1016/j.neuropharm.2013.08.008

[4] Fang L, Wang YN, Cui XL, Fang SY, Ge JY, Sun Y, Liu ZH 2012 The role and mechanism of action of activin A in neurite outgrowth of chicken embryonic dorsal root ganglia. J. Cell Sci. 125, 1500–1507. (doi:10.1242/jcs.094151) 22275431 10.1242/jcs.094151

[5] Dean M, Davis DA, Burdette JE 2017 Activin A stimulates migration of the fallopian tube epithelium, an origin of high-grade serous ovarian cancer, through non-canonical signaling. Cancer Lett. 391, 114–124. (doi:10.1016/j.canlet.2017.01.011) 28115208 10.1016/j.canlet.2017.01.011 PMC5336484

[6] Aleman-Muench GR, Soldevila G 2012 When versatility matters: activins/inhibins as key regulators of immunity. Immunol. Cell Biol. 90, 137–148. (doi:10.1038/icb.2011.32) 21537340 10.1038/icb.2011.32

[7] Wu S, Qi Y, Niu LM, Xie DX, Cui XL, Liu ZH 2015 Activin A as a novel biomarker for colorectal adenocarcinoma in humans. Eur. Rev. Med. Pharmacol. Sci. 19, 4371–4378. 26636525

[8] Ling N, Ying SY, Ueno N, Shimasaki S, Esch F, Hotta M, Guillemin R 1986 Pituitary FSH is released by a heterodimer of the beta-subunits from the two forms of inhibin. Nature 321, 779–782. (doi:10.1038/321779a0) 3086749 10.1038/321779a0

[9] Kingsley DM 1994 The TGF-beta superfamily: new members, new receptors, and new genetic tests of function in different organisms. Genes Dev. 8, 133–146. (doi:10.1101/gad.8.2.133) 8299934 10.1101/gad.8.2.133

[10] Jones CP, Gregory LG, Causton B, Campbell GA, Lloyd CM 2012 Activin A and TGF-beta promote T(H)9 cell-mediated pulmonary allergic pathology. J. Allergy Clin. Immunol. 129, 1000–1010.e3. (doi:10.1016/j.jaci.2011.12.965) 22277204 10.1016/j.jaci.2011.12.965 PMC3385370

[11] Sampath K, Robertson EJ 2016 Keeping a lid on nodal: transcriptional and translational repression of nodal signalling. Open Biol. 6, 150200 (doi:10.1098/rsob.150200) 26791244 10.1098/rsob.150200 PMC4736825

[12] Al-Salihi MA, Herhaus L, Sapkota GP 2012 Regulation of the transforming growth factor beta pathway by reversible ubiquitylation. Open Biol. 2, 120082 (doi:10.1098/rsob.120082) 22724073 10.1098/rsob.120082 PMC3376735

[13] Massague J, Wotton D 2000 Transcriptional control by the TGF-beta/Smad signaling system. EMBO J. 19, 1745–1754. (doi:10.1093/emboj/19.8.1745) 10775259 10.1093/emboj/19.8.1745 PMC302010

[14] Fiocchi C 2001 TGF-beta/Smad signaling defects in inflammatory bowel disease: mechanisms and possible novel therapies for chronic inflammation. J. Clin .Invest. 108, 523–526. (doi:10.1172/JCI13863) 11518725 10.1172/JCI13863 PMC209413

[15] Takagi K et al. 2011 Activation of the activin A-ALK-Smad pathway in systemic sclerosis. J. Autoimmun. 36, 181–188. (doi:10.1016/j.jaut.2010.09.004) 21377836 10.1016/j.jaut.2010.09.004

[16] Beyrau M, Bodkin JV, Nourshargh S 2012 Neutrophil heterogeneity in health and disease: a revitalized avenue in inflammation and immunity. Open Biol. 2, 120134 (doi:10.1098/rsob.120134) 23226600 10.1098/rsob.120134 PMC3513838

[17] Nadkarni S et al. 2016 Neutrophils induce proangiogenic T cells with a regulatory phenotype in pregnancy. Proc. Natl Acad. Sci. USA 113, E8415–E8424. (doi:10.1073/pnas.1611944114) 27956610 10.1073/pnas.1611944114 PMC5206541

[18] de Oliveira S, Rosowski EE, Huttenlocher A 2016 Neutrophil migration in infection and wound repair: going forward in reverse. Nat. Rev. Immunol. 16, 378–391. (doi:10.1038/nri.2016.49) 27231052 10.1038/nri.2016.49 PMC5367630

[19] Kruger P et al. .2015 Neutrophils: between host defence, immune modulation, and tissue injury. PLoS Pathog. 11, e1004651 (doi:10.1371/journal.ppat.1004651) 25764063 10.1371/journal.ppat.1004651 PMC4357453

[20] Brazil JC, Parkos CA 2016 Pathobiology of neutrophil-epithelial interactions. Immunol. Rev. 273, 94–111. (doi:10.1111/imr.12446) 27558330 10.1111/imr.12446 PMC5000857

[21] Phillips DJ, de Kretser DM, Hedger MP 2009 Activin and related proteins in inflammation: not just interested bystanders. Cytok. Growth Factor Rev. 20, 153–164. (doi:10.1016/j.cytogfr.2009.02.007) 10.1016/j.cytogfr.2009.02.007 19261538

[22] Werner S, Alzheimer C 2006 Roles of activin in tissue repair, fibrosis, and inflammatory disease. Cytok. Growth Factor Rev. 17, 157–171. (doi:10.1016/j.cytogfr.2006.01.001) 10.1016/j.cytogfr.2006.01.001 16481210

[23] Lee JK et al. 2016 Serum activin-A as a predictive and prognostic marker in critically ill patients with sepsis. Respirology 21, 891–897. (doi:10.1111/resp.12751) 26969968 10.1111/resp.12751

[24] Linko R, Hedger MP, Pettila V, Ruokonen E, Ala-Kokko T, Ludlow H, de Kretser DM 2014 Serum activin A and B, and follistatin in critically ill patients with influenza A(H1N1) infection. BMC Infect Dis. 14, 253 (doi:10.1186/1471-2334-14-253) 24885241 10.1186/1471-2334-14-253 PMC4101860

[25] Ebert S, Nau R, Michel U 2010 Role of activin in bacterial infections: a potential target for immunointervention? Immunotherapy 2, 673–684. (doi:10.2217/imt.10.64) 20874651 10.2217/imt.10.64

[26] Apostolou E et al. 2012 Activin-A overexpression in the murine lung causes pathology that simulates acute respiratory distress syndrome. Am. J. Respir. Crit. Care Med. 185, 382–391. (doi:10.1164/rccm.201105-0784OC) 22161160 10.1164/rccm.201105-0784OC

[27] Sideras P, Apostolou E, Stavropoulos A, Sountoulidis A, Gavriil A, Apostolidou A, Andreakos E 2013 Activin, neutrophils, and inflammation: just coincidence? Semin. Immunopathol. 35, 481–499. (doi:10.1007/s00281-013-0365-9) 23385857 10.1007/s00281-013-0365-9 PMC7101603

[28] Ogawa K, Funaba M, Chen Y, Tsujimoto M 2006 Activin A functions as a Th2 cytokine in the promotion of the alternative activation of macrophages. J. Immunol. 177, 6787–6794. (doi:10.4049/jimmunol.177.10.6787) 17082592 10.4049/jimmunol.177.10.6787

[29] Wu H, Chen Y, Winnall WR, Phillips DJ, Hedger MP 2013 Regulation of activin A release from murine bone marrow-derived neutrophil precursors by tumour necrosis factor-alpha and insulin. Cytokine 61, 199–204. (doi:10.1016/j.cyto.2012.09.018) 23116663 10.1016/j.cyto.2012.09.018

[30] Mayadas TN, Cullere X, Lowell CA 2014 The multifaceted functions of neutrophils. Annu. Rev. Pathol. 9, 181–218. (doi:10.1146/annurev-pathol-020712-164023) 24050624 10.1146/annurev-pathol-020712-164023 PMC4277181

[31] Bardoel BW, Kenny EF, Sollberger G, Zychlinsky A 2014 The balancing act of neutrophils. Cell Host Microbe 15, 526–536. (doi:10.1016/j.chom.2014.04.011) 24832448 10.1016/j.chom.2014.04.011

[32] Segal AW 2016 NADPH oxidases as electrochemical generators to produce ion fluxes and turgor in fungi, plants and humans. Open Biol. 6, 160028 (doi:10.1098/rsob.160028) 27249799 10.1098/rsob.160028 PMC4892433

[33] Wilson KM, Smith AI, Phillips DJ 2006 Stimulatory effects of lipopolysaccharide on endothelial cell activin and follistatin. Mol. Cell. Endocrinol. 253, 30–35. (doi:10.1016/j.mce.2006.03.041) 16697104 10.1016/j.mce.2006.03.041

[34] Chen Y, Wu H, Winnall WR, Loveland KL, Makanji Y, Phillips DJ, Smith JA, Hedger MP 2011 Tumour necrosis factor-alpha stimulates human neutrophils to release preformed activin A. Immunol. Cell Biol. 89, 889–896. (doi:10.1038/icb.2011.12) 21445090 10.1038/icb.2011.12

[35] Bilezikjian LM, Turnbull AV, Corrigan AZ, Blount AL, Rivier CL, Vale WW 1998 Interleukin-1beta regulates pituitary follistatin and inhibin/activin betaB mRNA levels and attenuates FSH secretion in response to activin-A. Endocrinology 139, 3361–3364. (doi:10.1210/endo.139.7.6190) 9645713 10.1210/endo.139.7.6190

[36] Gonzalez-Dominguez E et al. 2016 Atypical Activin A and IL-10 production impairs human CD16^+^ monocyte differentiation into anti-inflammatory macrophages. J. Immunol. 196, 1327–1337. (doi:10.4049/jimmunol.1501177) 26729812 10.4049/jimmunol.1501177

[37] Abe M, Shintani Y, Eto Y, Harada K, Kosaka M, Matsumoto T 2002 Potent induction of activin A secretion from monocytes and bone marrow stromal fibroblasts by cognate interaction with activated T cells. J. Leukoc. Biol. 72, 347–352. 12149426

[38] Cho SH, Yao Z, Wang SW, Alban RF, Barbers RG, French SW, Oh CK 2003 Regulation of activin A expression in mast cells and asthma: its effect on the proliferation of human airway smooth muscle cells. J. Immunol. 170, 4045–4052. (doi:10.4049/jimmunol.170.8.4045) 12682233 10.4049/jimmunol.170.8.4045

[39] Kolaczkowska E, Kubes P. 2013 Neutrophil recruitment and function in health and inflammation. Nat. Rev. Immunol. 13, 159–175. (doi:10.1038/nri3399) 23435331 10.1038/nri3399

[40] Inoue A *et al* 2016 TIARP attenuates autoantibody-mediated arthritis via the suppression of neutrophil migration by reducing CXCL2/CXCR2 and IL-6 expression. Sci. Rep. 6, 38684 (doi:10.1038/srep38684) 27995997 10.1038/srep38684 PMC5171802

[41] de Kretser DM, Bensley JG, Phillips DJ, Levvey BJ, Snell GI, Lin E, Hedger MP, O'Hehir RE 2016 Substantial increases occur in serum activins and Follistatin during lung transplantation. PLoS ONE 11, e0140948 (doi:10.1371/journal.pone.0140948) 26820896 10.1371/journal.pone.0140948 PMC4731072

[42] Li N, Cui X, Ge J, Li J, Niu L, Liu H, Qi Y, Liu Z, Wang Y 2013 Activin A inhibits activities of lipopolysaccharide-activated macrophages via TLR4, not of TLR2. Biochem. Biophys. Res. Commun. 435, 222–228. (doi:10.1016/j.bbrc.2013.04.077) 23665022 10.1016/j.bbrc.2013.04.077

[43] Ge J, Wang Y, Feng Y, Liu H, Cui X, Chen F, Tai G, Liu Z 2009 Direct effects of activin A on the activation of mouse macrophage RAW264.7 cells. Cell. Mol. Immunol. 6, 129–133. (doi:10.1038/cmi.2009.18) 19403063 10.1038/cmi.2009.18 PMC4002650

[44] Zhang XJ, Li Y, Tai GX, Xu GY, Zhang PY, Yang Y, Lao FX, Liu ZH 2005 Effects of activin A on the activities of the mouse peritoneal macrophages. Cell. Mol. Immunol. 2, 63–67. 16212913

